# Integrated metabolomics and metagenomics uncover pathogenic mechanisms of Fusarium wilt and faba bean defense responses

**DOI:** 10.1038/s41538-025-00673-8

**Published:** 2026-01-19

**Authors:** Jiaqi Zheng, Chaowen Zhang, Siheng Xiang, Mengqing Li, Hongji Wang, Kexin Shi, Dorjeeh Tondrob, Yuzhu Han

**Affiliations:** 1 College of Animal Science and Technology, Southwest University, Chongqing, China; 2 Department of Food Science and Nutrition, The Hong Kong Polytechnic University, Hong Kong, Hong Kong SAR, China; 3 Chongqing University Herbivore Engineering Research Center, Chongqing, China; 4 Institute of Biotechnology, Zhejiang University, Hangzhou, China; 5 Improvement, Institute of Pratacultural Science, Xizang Academy of Agricultural and Animal Husbandry Sciences, Lhasa, China

**Keywords:** Microbiology, Plant sciences

## Abstract

Fusarium wilt diseases pose a huge threat to faba bean (*Vicia faba* L.) production globally, with significant outbreaks in Chongqing, China. Symptomatic plants showed wilting leaves and rotten roots, ultimately perishing in the advanced stage. Morphological features, multilocus phylogenetic analyses, and pathogenicity tests demonstrated that the primary causal agent was *Fusarium oxysporum*. Untargeted metabolomics of faba beans revealed substantial metabolic differences in the infected faba bean roots. Plants responded to fungal biotic stress by reprogramming key metabolic pathways, including alanine, aspartate, and glutamate metabolism, the citrate cycle, arginine biosynthesis, and jasmonic acid metabolism, which collectively underscore activated defense responses. Metagenome sequencing showed that Fusarium wilt significantly reshaped the structure of the rhizosphere microbiota and affected the abundance of genes encoding element cycling in soil. This work elucidates the pathogenic mechanisms of *F. oxysporum* by integrating pathogen identification, host metabolism, and microbiome ecology. Our findings offer biomarkers for disease diagnosis and targets for biocontrol, advancing sustainable management of Fusarium wilt diseases in legumes.

## Introduction

Faba bean (*Vicia faba* L.), a major legume crop with a long cultivation history in China, serves as a vital source of protein, vitamins, and amino acids for both livestock and humans. Beyond its nutritional value, faba bean enhances the sustainability of agroecosystems through increasing soil fertility and fixing biological nitrogen^1^. The crop demonstrates formaldehyde absorption capacity, suggesting potential applications in indoor air purification^2^ and ecological remediation^3^. Furthermore, faba bean plays a meaningful role in medicine as it can be used as a remedy for various physical disorders, such as cardiovascular and neurodegenerative diseases, and it is a promising candidate for anticancer and antidiabetic activity^4^.

*Fusarium oxysporum* is a ubiquitous soil-borne pathogen that invades a broad range of plant hosts, which induces diseases from roots and causes devastating vascular wilt, seriously lowering forage production and degrading vegetation’s nutritional value^5,6^. This species exerts an adverse impact not just on agriculture and economy, but also prompts mycosis infection in both humans and animals^7^. The pathogenicity is driven by diverse virulence factors, such as cell wall-degrading enzymes (CWDEs), mycotoxins, and secreted effectors, which enable host tissue invasion and colonization^8^. *F. oxysporum* is the primary pathogen of Fabaceae because it can infect plants through wounds, natural openings, or even intact roots^9^. In Southwest China, faba beans are cultivated on a vast scale, and Fusarium wilt disease (FWD) is a severe and recurrent challenge that leads to significant economic losses every year^10,11^.

Plants produce a wide variety of secondary metabolites that are involved in different functions for environmental adaptation, including communication through signaling molecules, stress tolerance, and chemical barriers to protect them against pathogen attack. Phytohormones such as jasmonic acid (JA), salicylic acid (SA), abscisic acid (ABA), and ethylene (ET) function as universal signaling hubs that enable plants to perceive stress and initiate differentiated physiological and metabolic responses^12^. Metabolomics provides a powerful approach to monitor dynamic changes in plant metabolite levels during growth, development, and environmental adaptation. Such information offers researchers critical insights into plant adaptation mechanisms under adverse conditions and facilitates the development of strategies to enhance stress tolerance. Several studies have investigated metabolic alterations in legumes under biotic stresses induced by fungi, oomycetes, and pests^13–15^, as well as subjected to abiotic stresses such as heavy metal contamination^16^.

Microbial community assembly and plant evolution are controlled by complex interactions between microbes, plant hosts, and the environment, and the resilience and assembly of this system are dynamically altered when plants encounter stress^17,18^. The microbiome around plant rhizosphere plays an important role in plant health, productivity, and ecosystem function^19^. Upon pathogen infection, plants undergo immune responses triggered by pathogen-derived molecules, leading to alterations in root exudate composition. These changes subsequently modulate the rhizosphere microbiome, potentially enriching beneficial microorganisms that contribute to plant defense^20,21^. In recent years, integrated analysis of metabolomic and microbiome data has provided deeper insights into how plant growth and adaptive processes shape the structure and function of microbial communities.

In this study, we identified *F. oxysporum* as the pathogenic fungi responsible for faba bean wilt disease in Chongqing, China, and profiled the associated metabolic and microbial community changes during infection using metabolomics and metagenomics approaches. To our knowledge, this is the first report of *F. oxysporum* causing FWD on faba bean in the Chongqing region. Our findings elucidate the intricate metabolic reprogramming in faba bean during pathogen attack and decipher key interactions within the legume microbiome, offering a foundation for future strategies aimed at harnessing microbial communities to improve crop health and productivity.

## Results

### **The pathogen was identified as*Fusarium oxysporum*based on morphological characterization and phylogenetic analyses**

**Fig. 1:**
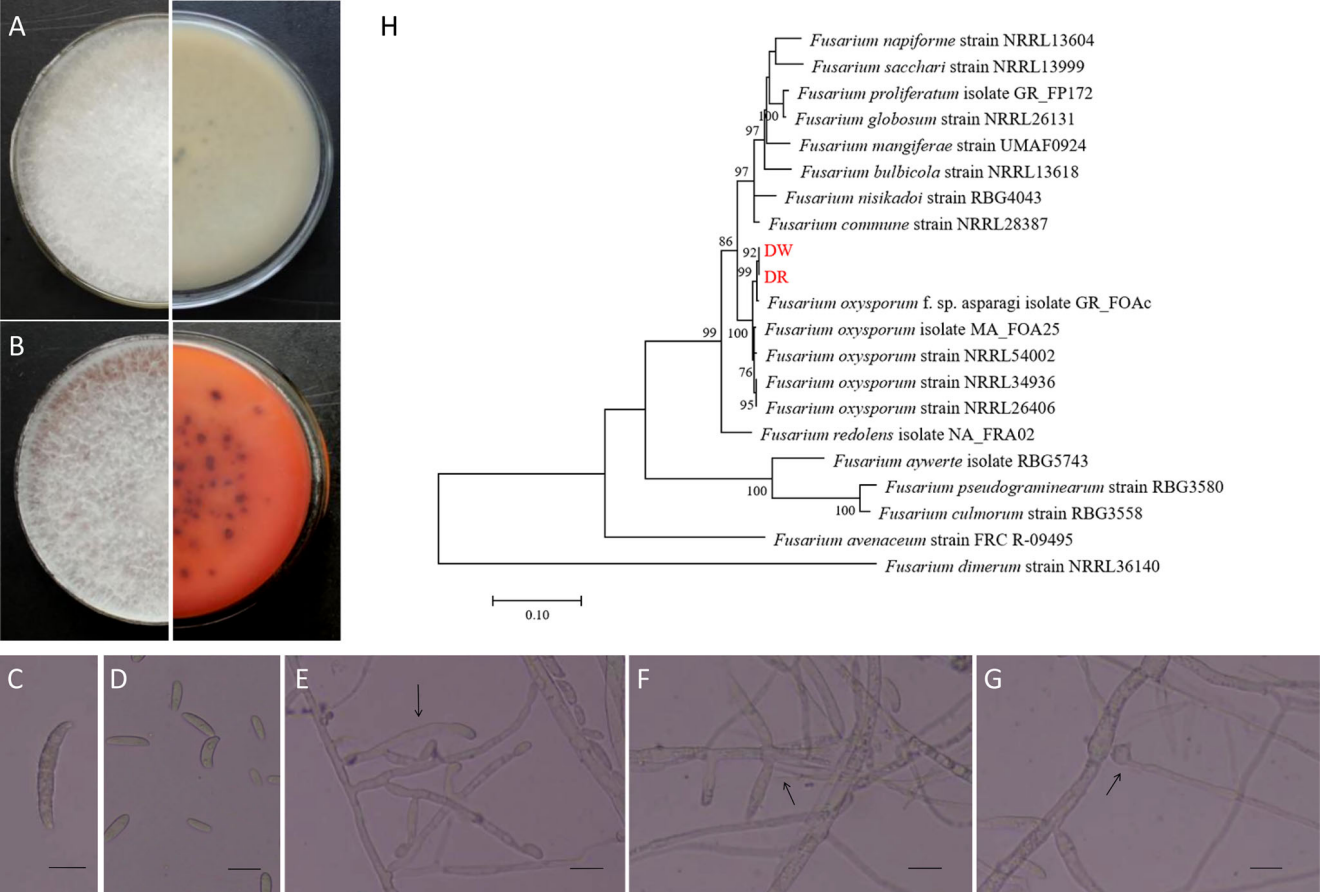
Morphology of *Fusarium oxysporum* isolated from faba bean infected with FWD. **A**, **B** The upper surface and the lower surface of the fungal culture grown on PDA for 25 days. Falcate-shaped macroconidia (**C**), microconidia (**D**), monophialides produce microconidia or macroconidia (**E**), branched conidiophores (**F**), and chlamydospores (**G**). Scale bars in **C**–**G** = 10 μm. **H** Maximum-Likelihood tree based on a combined dataset of the translation elongation factor 1-alpha (EF1-α) and the second largest subunit of RNA polymerase II (rpb2) region. The scale bar indicates 0.1 nucleotide substitutions.

The average disease incidence and index of wilting symptom on faba beans in Beibei and Rongchang District in Chongqing were recorded as 83.90% and 79.20 ± 4.12, respectively. Typical disease symptoms include wilting leaves, browning stems, and rotten roots. A total of 19 isolates of *Fusarium*-like fungi were isolated from rotten tissues of the faba bean. The colony surface was cottony, white, with a filamentous margin, and the reverse sides were uniformly white to brownish red (Fig. 1A, B). Aerial mycelia were abundant, densely floccose to fluffy, accompanied by high sporulation. Most macroconidia were falcate, slender, usually with 5–7 septate, featured apical cells slightly curved and foot-shaped basal cells. They were variable in size, measuring 16.7–41.2 × 2.4–5.8 μm (Fig. 1C). Microconidia were oval to cylindrical, with flattened bases and were mostly formed in a false head, with 0–1 septa, and 3.7–12.2 × 2.1–3.8 μm in size (Fig. 1D). Monophialides and polyphialides were elongate-ampulliform to subcylindrical with apical tapering, or short and ossiform with broader basal regions (Fig. 1E). Aerial conidiophores were abundant, of a size of 6.7–20.4 × 1.6–2.4 μm, branched sparsely or formed laterally (Fig. 1F). Chlamydospores were globose to sub-globose in shape and produced terminally, single or in pairs, measuring 4.8–15.1 μm in diameter (Fig. 1G).

PCR amplification of the ITS, TUB2, EF1-α, mtSSU, rpb2, and GAPDH regions yielded amplicons of 475–487, 289–303, 260–264, 673–680, 1015–1018, and 1080–1131 bp, respectively. The obtained sequences of the representative strain DR and DW were deposited in GenBank with the following accession numbers: OL518958–OL518959 (ITS), OL551680–OL551681 (TUB2), OM238215–OM238216 (EF1-α), OM257406–OM257407 (mtSSU), OM287406–OM287407 (rpb2), and OM287408–OM287409 (GAPDH). Based on BLAST searches, all sequences showed high similarity (96.96–100%) to related *F. oxysporum* species.

EF-1α is the most recommended locus for its high informativeness in inferring the identity of *Fusarium* species^22^, followed by the rpb2 region, which is useful for resolving distinctions across or beyond the *Fusarium* species level^23^. The combined EF-1α and rpb2 datasets provided an appropriate resolution of phylogeny^24^. Nucleotide sequences of reference taxa were obtained from GenBank (Supplementary Table 4). The Maximum-Likelihood tree concatenated of EF1-α and rpb2 regions was shown in Fig. 1H, which revealed that the DR and DW isolates were clustered together with a 96% bootstrap value. These two strains were grouped in the same clade with *F. oxysporum*.

### Pathogenicity tests and plant defense responses to Fusarium infection

**Fig. 2:**
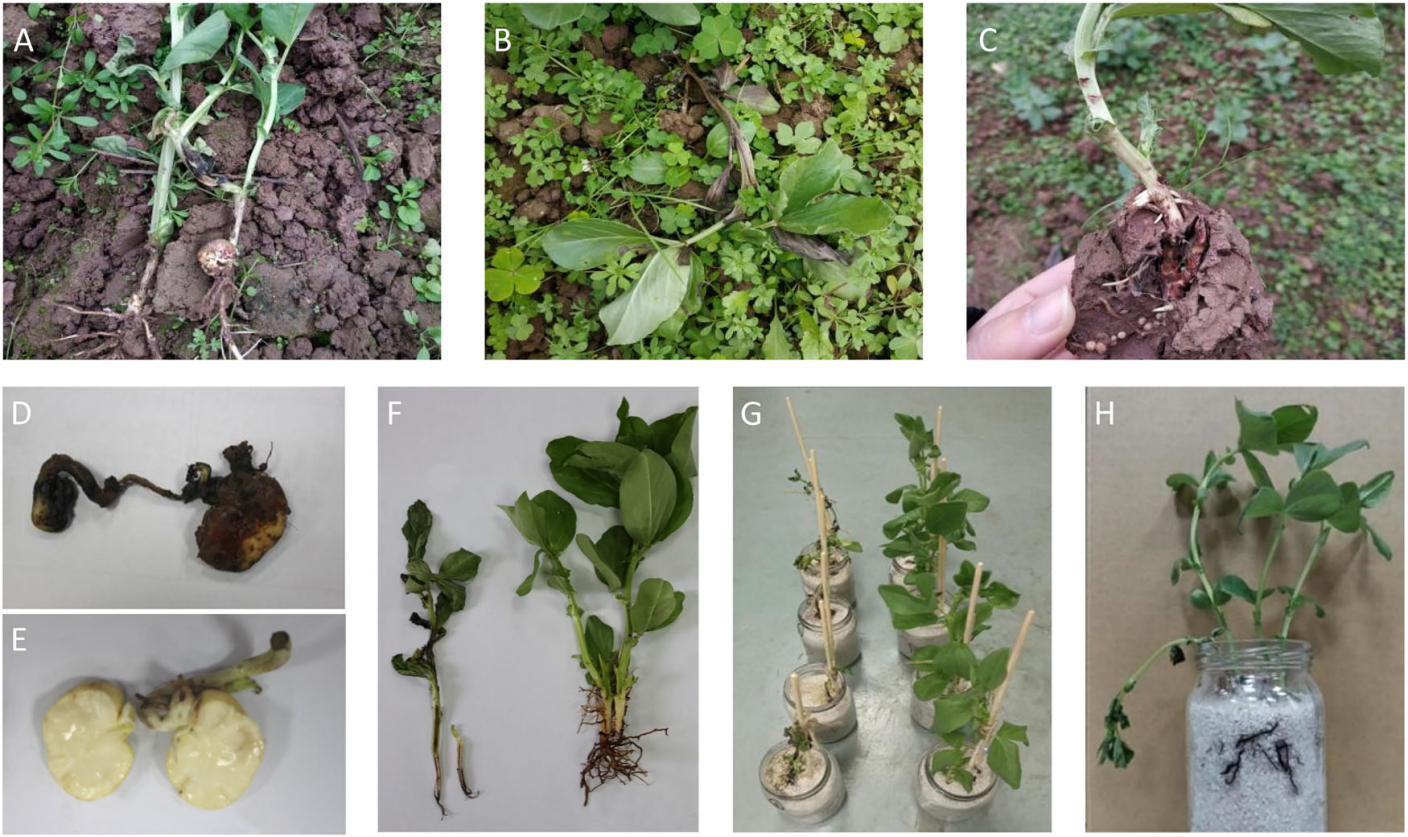
Disease progression and symptom development of Fusarium wilt in faba beans. **A**–**C** Typical Fusarium wilting disease symptom on faba beans observed in the field. Rotten (**D**) and healthy (**E**) faba beans collected from the field. **F**, **G** Wilting and stunting symptoms of faba bean inoculated with *Fusarium oxysporum* isolate (left) compared with controls (right). Infected faba bean (**H**) showed wilting and root rot symptoms.

The pathogenicity assays demonstrated *F. oxysporum* isolates DW, which have been submitted to the China General Microbiological Culture Collection Center with collection ID of CGMCC3.23786, were hazardous to faba bean, generating similar disease symptoms as natural onsets in the field. After 7 days of inoculation, the typical symptoms of infected plants included stunted growth, discolored leaves, and black necrotic lesions formed from the base of the stem. The disease index in the pot experiments was recorded as 37.14 ± 2.33 in 30 days and 77.86 ± 3.11 in 60 days. *Fusarium* isolates caused dark lesions on the root system, and at a severe stage, the whole taproot decayed. The entire plants wilted and died in about 80 days, and the vascular tissues turned dark brown or black (Fig. 2). All Fusarium-inoculated plants exhibited wilting symptoms, while the control group remained healthy. The pathogens were re-isolated from the lesions and were morphologically and molecularly identical to *F. oxysporum*, thereby satisfying Koch’s postulates.

### Boosting plant resilience to pathogens through antioxidant system activation

**Fig. 3:**
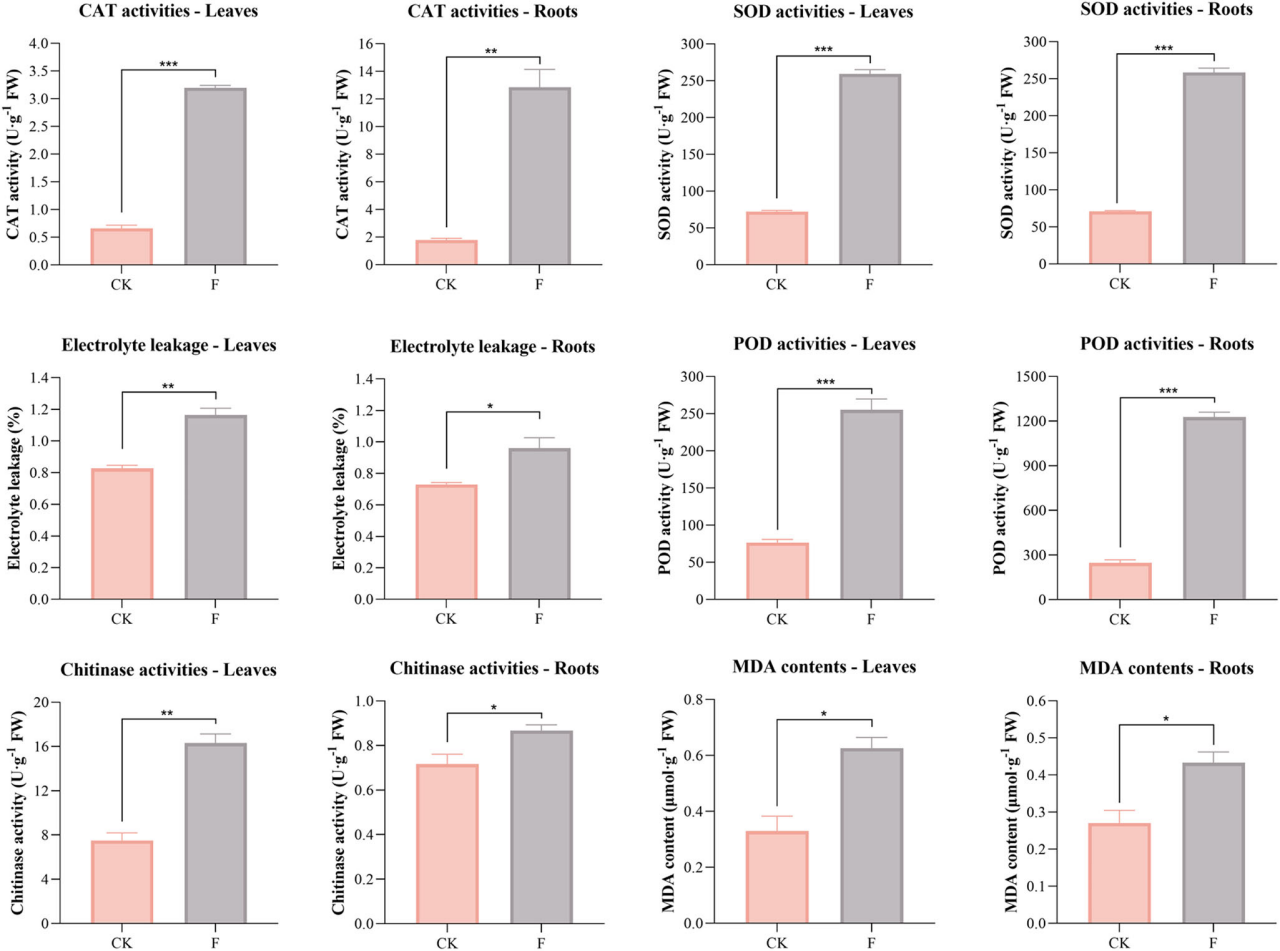
The CAT, SOD, POD, and chitinase activities, as well as the levels of electrolyte leakage and malondialdehyde (MDA) in the leaves and roots between healthy (CK) and Fusarium-infected (F) faba beans. FW refers to the fresh weight of plants. ^*^p < 0.05, ^**^p < 0.01, and ^***^p < 0.001.

Antioxidant enzymes in plant first-line defense, such as catalase (CAT), superoxide dismutase (SOD), and peroxidase (POD), which can help eliminate superoxide radicals and hydrogen peroxide, play a crucial role in mitigating oxidative stress and enhancing plant defense^25^. As shown in Fig. 3, faba beans infected with *F. oxysporum* exhibited a significant increase in antioxidant functions. For example, the SOD, CAT, and POD activity of faba beans subjected to pathogens increased by 2.64-fold, 6.15-fold, and 3.97-fold in root tissues, respectively, compared with the control. Leaves showed lower induction levels across antioxidant enzymes when compared to the control, with SOD improved by 2.58-fold, CAT by 3.84-fold, and POD by 2.33-fold.

Plant chitinases are significantly induced by pathogen attack, serving dual functions in defense and development^26,27^. Chitinase activity in the roots of diseased faba bean exhibited an evident rise by over onefold compared to the healthy plants, while foliar chitinase levels showed a rise of over twofold. Diseased leaves displayed obviously higher antioxidant capacity than controls, suggesting a robust physiological adaptation to pathogenic fungal invasion. The expression of root chitinases is regulated by rhizosphere microorganisms, which can either activate or suppress the synthesis of these enzymes^28^.

Membrane damage in plants was evaluated through malondialdehyde (MDA) production and electrolyte leakage. The accumulation of MDA in roots and leaves of faba bean infested with Fusarium was significantly higher (*p* < 0.05). This result indicated heightened lipid peroxidation, a hallmark of oxidative damage under biotic stress. Increased electrolyte leakage in both leaves and roots further corroborated membrane integrity disruption (*p* < 0.05), resulting from the plants bolstering their defense mechanisms in response to pathogen invasion. The rise in electrolyte leakage was more pronounced in the diseased leaves than in the roots. Overall, these findings demonstrate that Fusarium-infected plants enhance their defense response against pathogen invasion by enhancing antioxidant activity and inducing cellular damage.

### Fusarium wilt altered root metabolism in faba beans to resist pathogenic fungal biotic stresses

**Fig. 4:**
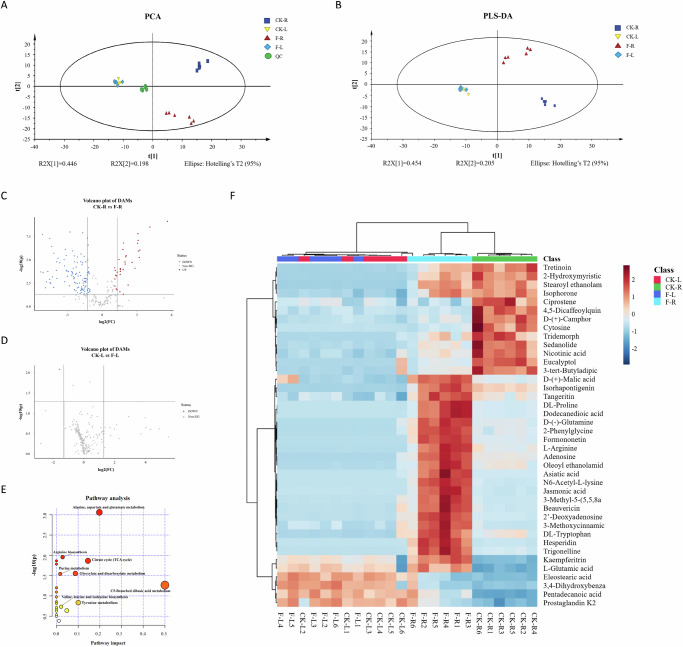
Metabolite profiles in roots and leaves of healthy and diseased plants. **A** Principal component analysis score plot (PCA). **B** Partial least squares discrimination analysis score plot (PLS-DA). **C**, **D** Volcano plot of differentially accumulated metabolites (DAMs) between Fusarium wilt and healthy faba bean in roots and leaves. *Y* axis: *p* value, *X* axis: fold change (FC). Red dots represent upregulated metabolites, blue dots show downregulated metabolites, and gray dots indicate metabolites that did not change. **E** Pathways analysis in the root. Each point represents a different metabolic pathway, and the size of the point represents the degree of change in each metabolic pathway. **F** Variation in metabolites between Fusarium wilt and healthy faba bean in roots and leaves in a heat map screened by ANOVA (*p* < 0.05). CK-R roots from healthy plants, F-R roots from diseased plants, CK-L leaves from healthy plants, F-L leaves from diseased plants, QC quality control samples.

The metabolite changes in leaves and roots between infected faba beans and the control group were determined by LC-MS and untargeted metabolomics analysis. A total of 246 metabolites were identified. SIMCA software was used to convert and classify the normalized data, which was then analyzed using principal component analysis (PCA) and partial least squares discrimination analysis score plot (PLS-DA). In the PCA score plot, two PCs accounted for more than 64.4% of the total variance (PC1 and PC2 explained 44.6% and 19.8%, respectively; Fig. 4A). All samples were aggregated in the 95% Hotelling’s T2 ellipse in the score plot, and then the QC samples were clustered in the middle, suggesting the metabolic approach had good stability^29^. In faba bean roots, there are remarkable changes between the plants infected with *F. oxysporum* (F-R) and the control group (CK-R). As shown in Fig. 4A, F-R was clearly separated from the CK-R group, however, leaves detached from symptomatic faba bean roots (F-L) were aggregated with the control group (CK-L), with no significant differences.

PLS-DA was used to determine the differences between the Fusarium-infected faba bean and control groups (Fig. 4B), which are consistent with the results of PCA score plots. The F-R and CK-R groups were clearly distinguished as their metabolites showed significant differences. In this model, permutation test cross-validation was performed 200 times to ensure the applicability of the model (*R*^2^*X*) with high predictability (*Q*^2^), while the value of *R*^2^*X* was 0.659 (close to 1) and *Q*^2^ was 0.587 (>0.5), respectively (Supplementary Fig. 2). The PLS-DA score plot showed a relatively good fit, reproducibility, and stability^30^. Even though *R*^2^ and *Q*^2^ had some overlap, the intercept of all permutation plots (<−0.335) indicated that the model was not over-fitting^31^.

Differentially accumulated metabolites (DAMs) were screened by fold change (FC) value > 1.5 combined with variable importance in projection (VIP) value > 1 and *p* value < 0.01^32^, and then they were uploaded into MetaboAnalyst 5.0 (https://www.metaboanalyst.ca/) for evaluation, combining topology with pathway enrichment analysis. The volcano diagram demonstrated that the changes of metabolic differentials in faba bean Fusarium wilt were primarily in the roots (Fig. 4C, D). As a result, the pathway enrichment analysis was mainly conducted based on the roots. Compared with the CK group, the major pathway alterations associated with faba bean Fusarium wilt involved alanine, aspartate and glutamate metabolism, arginine biosynthesis, and the citrate cycle (TCA cycle; Fig. 4E).

### Fusarium wilt reshaped microbial diversity and community composition in the faba bean rhizosphere

**Fig. 5:**
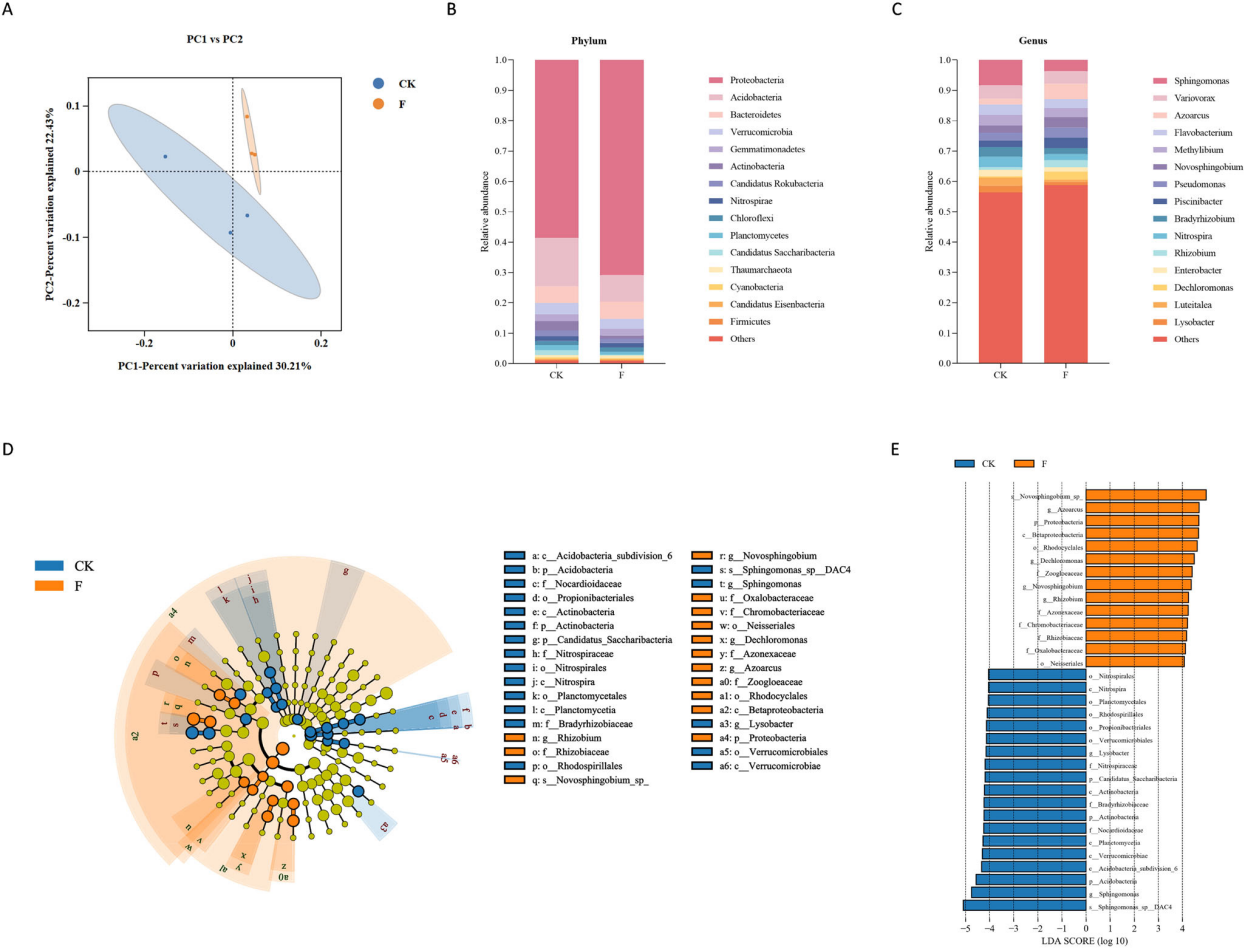
Rhizosphere microbiome restructuring in faba beans under *Fusarium oxysporum* infection. **A** Principal coordinate analysis (PCoA) of beta diversity was measured based on binary Jaccard distance at the genus level. **B**, **C** Relative abundances of the bacterial community at the phylum and genus levels collected from Fusarium-infected and healthy faba bean rhizosphere. **D** Cladogram of microbial communities with significant differences between CK and F groups based on the linear discriminant analysis effect size analysis (LEfSe). CK represents the control group, and F represents microbial communities of faba beans infected with Fusarium wilt. **E** LDA scores of the differentially abundant taxa of the Fusarium-infected group compared with the control group.

The beta diversity of the rhizosphere microbiota was assessed using PCoA based on binary Jaccard dissimilarity distances between the CK and F groups. PCoA1 (22.43%) and PCoA2 (30.21%) described a total of 52.64% variation in bacterial community composition. The PCoA analysis revealed a clear separation between the control and the Fusarium-exposed groups, indicating that the infection in faba bean altered the structure of the microbial community (Fig. 5A). PERMANOVA test of beta diversity confirmed a significant difference between the groups (*R*^2^ = 0.246, *p* value < 0.01; Supplementary Fig. 3).

The microbiota at the kingdom, phylum, and genus level in the faba bean rhizosphere are shown in Figure 5B, C, and Supplementary Fig. 4. The taxonomical classification of metagenomic sequencing data for the sample processed showed the dominance of bacteria (>89.50%), followed by archaea (>0.56%), viruses (>0.13%), and fungi (>0.03%). The proportion of viruses increased while the proportion of fungi decreased when the plant was exposed to *F. oxysporum*. *Proteobacteria* (>56.05%), *Acidobacteria* (>6.23%), *Bacteroidetes* (>2.85%), and *Verrucomicrobia* (>2.81%) were the most abundant phyla with different relative abundances in all samples. *Proteobacteria* accounted for a large part of the rhizosphere community, which was then enriched after inoculation with pathogenic isolates (*p* < 0.05). The abundance of *Acidobacteria*, *Actinobacteria*, and *Candidatus Saccharibacteria* in healthy faba beans was higher than in the infected ones (*p* < 0.05). At the genus level, with inoculation of *F. oxysporum*, the relative abundances of *Bradyrhizobium* and *Dechloromonas* increased, whereas the relative abundances of *Sphingomonas*, *Luteitalea*, and *Lysobacter* decreased (*p* < 0.05).

Microbial community analysis revealed significant diversity in rhizosphere composition between CK and Fusarium-infected treatments across all taxonomic levels (class to species). The differences in taxa among the treatments were shown within the cladogram representation generated via LEfSe, which unveiled the existence of 33 discriminative features between the healthy and the Fusarium-infected faba beans (LDA score > 4) (Fig. 5D, E). In the healthy group, the most pronounced shifts in the relative abundance of phylotypes between the infected plants were found for bacteria of the genus *Rhizobium* (phylum *Proteobacteria*—class *Alphaproteobacteria*—order *Rhizobiales*—family *Rhizobiaceae*), *Novosphingobium* (phylum *Proteobacteria*—class *Alphaproteobacteria*—order *Sphingomonadales*—family *Sphingomonadaceae*), *Dechloromonas* (phylum *Proteobacteria*—class *Betaproteobacteria*—order *Rhodocyclales*—family *Azonexaceae*), and *Azoarcus* (phylum *Proteobacteria*—class *Betaproteobacteria*—order *Rhodocyclales*—family *Zoogloeaceae*).

### Functional pathways affected by the Fusarium infection in faba beans

**Fig. 6:**
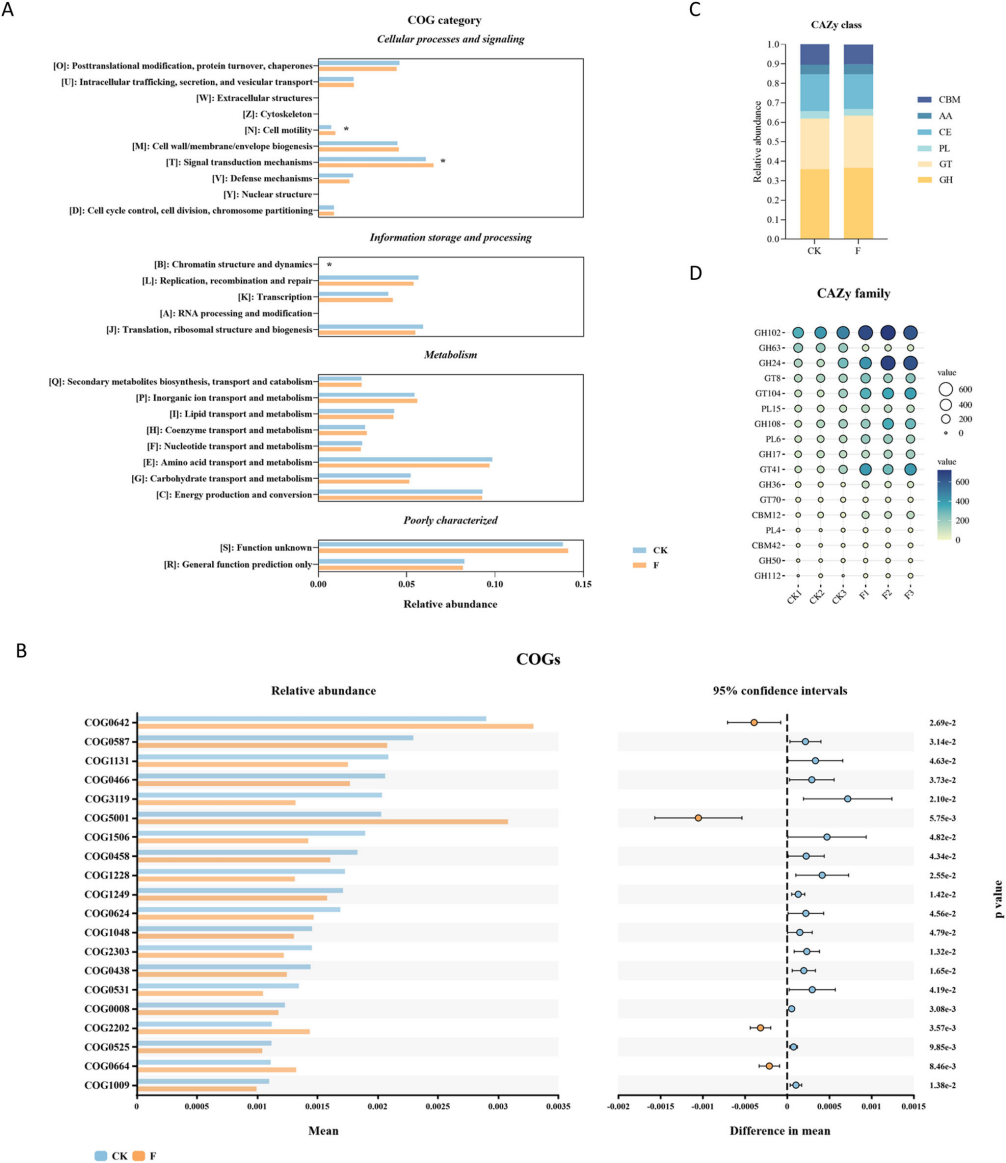
Functional profile of the rhizosphere microbiome in healthy and Fusarium-infected faba beans. **A** The functional annotation of faba bean rhizosphere microbiota genes by eggCOG. **B** Differential abundance of COGs between CK and F groups (*p* < 0.05). **C** Relative abundance of different CAZy classes in CK and F groups. **D** Differential abundance of CAZy families between CK and F groups (*p* < 0.05).

Based on the eggNOG analysis, the enrichment of proteins of both CK and F samples in categories mainly includes amino acid transport and metabolism (E), energy production and conversion (C), signal transduction mechanisms (T), translation and ribosomal biogenesis (J), inorganic ion transport and metabolism (P), replication, recombination, and repair (L), carbohydrate transport and metabolism (G), posttranslational modification, protein turnover, chaperones (O), and cell wall/membrane biogenesis (M) (Fig. 6A). The marked increased COGs in infected plants associated with signal transduction mechanisms (T), cell motility (N) and chromatin structure and dynamics (B) (*p* < 0.05), indicating an enhanced microbial adaption against stress.

A significant elevation was observed in the abundance of histidine kinase (COG0642), cyclic di-GMP metabolism protein (COG5001), and CRP/FNR family transcriptional regulator (COG0664) when faba bean responded to pathogen infection (Fig. 6B), which suggests the activation of the core regulatory machinery for sensing and responding to environmental stress. However, the relative abundance of COGs associated with energy metabolism and biosynthetic processes was markedly reduced in diseased plants, including DNA polymerase III (COG0587), tRNA synthetase (COG0008 and COG0525), NADH: ubiquinone oxidoreductase subunit (COG1009), galactosyltransferase (COG0438), and carbamoyl-phosphate synthetase (COG0458).

Carbohydrate-active enzymes (CAZy) are divided into six major groups, including glycosyltransferases (GTs), glycoside hydrolases (GHs), carbohydrate esterases (CEs), auxiliary activities (AAs), carbohydrate-binding modules (CBMs), and polysaccharide lyases (PLs). Among these, the relative abundance of CEs was lower in infected samples compared to the control (*p* < 0.05). In contrast, specific families of GHs (GH17, GH24, GH36, GH50, GH102, and GH112) and GTs (GT8, GT41, GT70, GT104, and GT108) were significantly upregulated in the infected group (Fig. 6C, D).

### Carbon, phosphorus, and sulfur cycling in the rhizosphere of Fusarium-infected faba beans

Metagenomic sequencing reveals that FWD significantly impacts the abundance of functional genes related to carbon (C), phosphorus (P), and sulfur (S) cycling in faba bean rhizosphere.

**Fig. 7:**
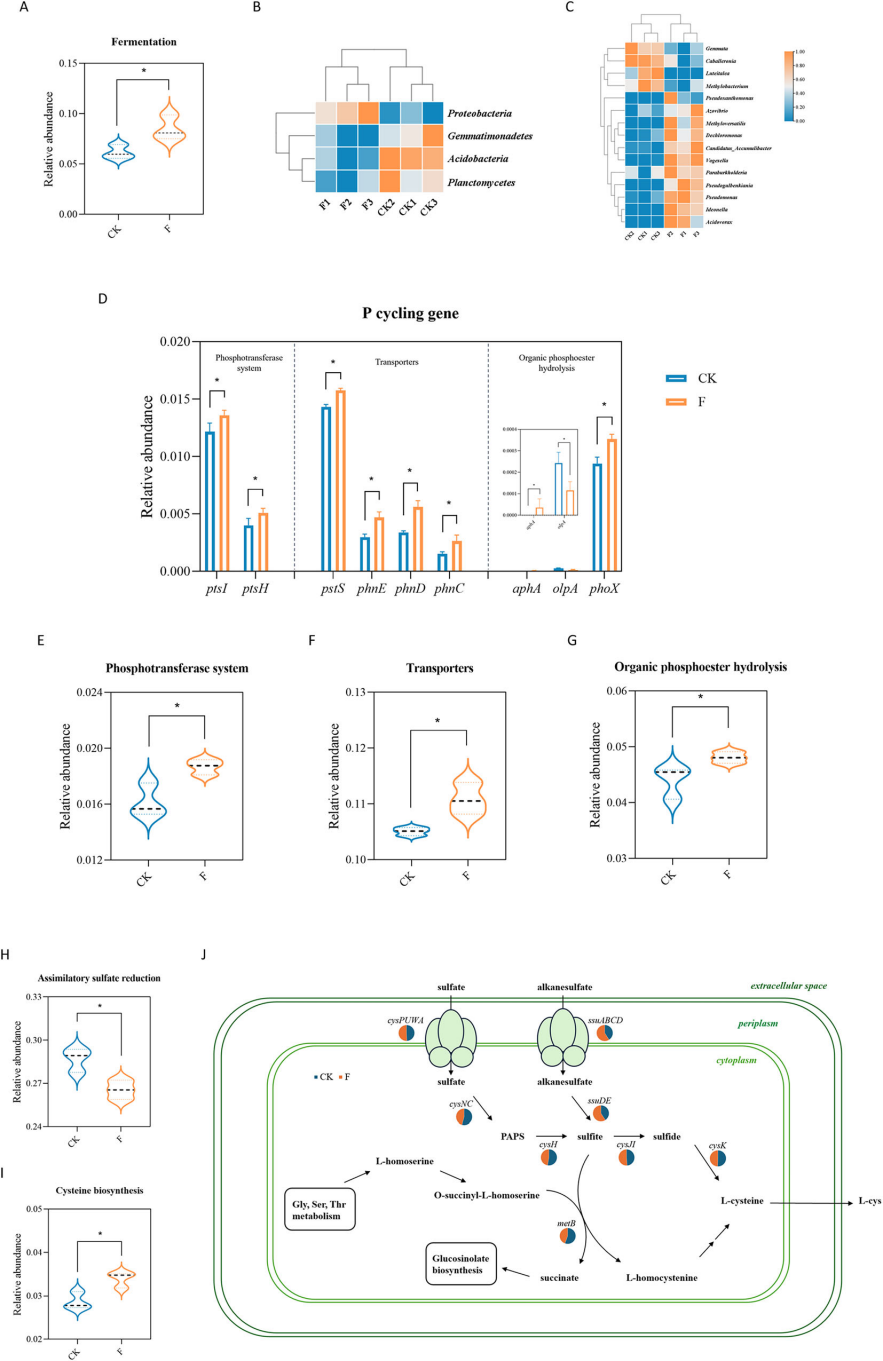
Microbial-driven biogeochemical cycling shifts in the faba bean rhizosphere under Fusarium infection. **A** Differentiate the classification of steps in the carbon cycle. Heatmaps of different microbial phyla (**B**) and genus (**C**) in the fermentation process. **D** The relative abundance of different genes in the phosphotransferase system, transporters, and organic phosphoester hydrolysis processes. **E**–**G** Differentiate the classification of steps in the phosphorus cycle. **H**, **I** Differentiate the classification of steps in the sulfur cycle. **J** Diagram of assimilatory sulfate reduction and cysteine biosynthesis pathway. The pie charts represent the relative abundance of genes involved in sulfur metabolism.

In the carbon cycling process, the abundance of soil carbon fermentation was significantly enriched in the rhizosphere community of infected faba beans (Fig. 7A). Pathogen infestation leads to root rot and reduced root respiration, while the rapid proliferation of pathogenic bacteria and accompanying microorganisms consumes large amounts of oxygen, resulting in an anoxic microenvironment at the inter-root level. In this condition, Fusarium triggered the fermentation metabolic pathway, converting sugars into products such as ethanol, lactic acid, and acetic acid. These metabolites not only acidify the inter-root environment but also inhibit the growth of beneficial microorganisms (e.g., plant-growth-promoting bacteria *Methylobacterium* and *Caballeronia*)^33–35^, further entrenching the ecological niche of Fusarium (Fig. 7B, C).

The most significant changes in the data set referred to microbial phosphate uptake and regulation systems, including transporters and organic phosphoester hydrolysis (Fig. 7E–G). Genes coding for highly efficient phosphate-specific transporter (e.g., *pstS*) and phosphonate transporter (*phnCDE*) showed a higher abundance in the rhizosphere of Fusarium-infected faba beans compared with the healthy group (Fig. 7D). The hydrolysis of organic phosphate esters by enzymes, such as alkaline phosphatase gene *phoX*, represents a crucial pathway for microbial phosphorus assimilation^36^. The abundance of these genes was higher in the infected group, leading to plant phosphorus starvation and weakened disease-suppressive functions^37^. After being infected with Fusarium, the gene abundance in the pathways of the phosphotransferase system (PTS) significantly increased (*p* < 0.05). PTS is primarily involved in the uptake of various carbohydrates and regulation of microbial metabolism, which couples with virulence and other functions^38,39^. Therefore, the higher abundance of *ptsI* and *ptsH* in the infected group indirectly enhances pathogen invasion.

Compared with healthy faba beans, the rhizosphere microbiota of infected plants had a significantly lower relative abundance of assimilatory sulfate reduction but a higher relative abundance of the cysteine biosynthesis process (Fig. 7H, I). This result suggests that Fusarium destroys the root system, inhibits sulfate uptake by plants, and reduces sulfur assimilation. As illustrated in Fig. 7J and Supplementary Fig. 5, plants upregulate cysteine synthase and utilize other sulfide-producing pathways to rapidly synthesize cysteine, which plays essential roles in defending against pathogen attack and responding to various biotic stresses^40,41^.

## Discussion

*Vicia* species are affected by several fungal diseases, and FWD is one of the most destructive diseases throughout the world. The rotten symptoms are caused by a complex of several fungal pathogens, including *Fusarium* spp., *Phytophthora* spp., *Pythium* spp., *Rhizoctonia* spp., *Aphanomyces* spp., and *Macrophomina* spp., which severely hinder agricultural production and hasten meadows vanishing. Faba bean FWD is a long-standing issue for Chinese growers. During colonization of host plants, *F. oxysporum* secretes a diverse repertoire of effector molecules into xylem vessels that operate in both apoplastic and intracellular compartments to facilitate fungal pathogenicity^42^. The *Secreted in Xylem* (*SIX*) protein family comprises crucial determinants of disease development, with specific members governing host range specificity across different *forma specialis* of this pathogen^43^. Notably, amplification of the *SIX6* gene was observed in isolates obtained from soybean and common bean with severe wilt symptoms, suggesting its potential role as a key virulence factor in Fabaceae-infecting strains^44,45^.

Since *F. oxysporum* induces tricky soil-borne disease, our study explored the pathogenesis of faba bean FWD and laid the groundwork for disease control and prevention. After crops are afflicted with FWD, their metabolic compounds serve as a signal to observe plants’ response against the pathogen, which helps to reveal the mechanisms of crop-pathogen interactions^46^. Based on metabolomics analysis, we found that faba bean roots and leaves produced remarkably varied metabolic compounds. Compared with the control, differential metabolites were generated in the infected roots. In contrast, few distinct metabolites were detected in the leaf parts. A probable factor could be that *F. oxysporum* directly invaded the plant root wound and contributed to interactions between the host plant and fungal pathogen. Researchers have found that transcriptome analysis of mung bean (*Vigna radiata* L.) rotten roots yielded 453 differentially expressed genes^47^. The root lies in the linkage between *F. oxysporum* and the plant. To resist the invasion of the pathogenic fungi, the plants would induce a series of defense responses, such as upregulation of the peroxidase gene (PvPOX1) to boost plant resistance to wilt^48^. Furthermore, plants could produce compounds that induce plant disease resistance, such as SA, JA, ferulic acid (FA), phenols, and flavonoids^49,50^. Part of the reason for this discovery was that faba bean samples suffering from Fusarium wilt may not achieve a sufficient disease incidence level, and the phytopathogenic fungi in the roots have not yet infested the overground parts. Hence, the leaves did not produce significant differential metabolites. Cuperlovic-Culf and his colleagues used NMR for non-targeted metabolomic analysis. They found that the above-ground parts of wheat samples infected with *F. oxysporum* for 48 h showed no significant changes in metabolic differentials, in alignment with our results^51^.

The differential metabolites released in the diseased site varied considerably from those in the CK group. The amino acid metabolites in the infected group showed significant elevations in glutamic acid, arginine, and proline. It should be noted that glutamic acid plays a crucial role in plant amino acid metabolism and is involved in the host defense response to pathogen infection as a signal between biotic and abiotic stresses^52^. When exposed to adversity, plants can regulate nitrogen and amino acid metabolism by secreting glutamic acid and enhance plant growth by restoring nitrogen balance. As illustrated in Fig. 4E, the significant enrichment of alanine, aspartate and glutamate metabolism together with TCA cycle, indicating a coordinated activation of amino acid and central energy metabolism. These metabolic adjustments suggest that glutamic acid metabolism, coupled with enhanced TCA cycle activity, was preferentially employed to counteract pathogenic fungal invasion in the roots of diseased faba beans. Dias and others revealed that using glutamic acid can enhance the resistance of rice leaf blast disease caused by *Pyricularia oryzae*^53^. Arginine is the most widely employed amino acid in plants and animals, and it engages in multiple metabolic processes in the body. Arginine and its secondary metabolite NO, in particular, play a huge part in plant stress resistance^54^. For example, arginase is a JA-induced protein^55^, and it is regarded as a precursor species of proline. In this work, we can observe that proline was significantly upregulated in diseased faba beans. Since substantial proline buildup often occurs when plants respond to the pathogenic attack, this was consistent with our results. Proline produced by plants was conducive to scavenging reactive oxygen species (ROS), maintaining cell homeostasis, water absorption, and osmoregulation^56^. In the heat map, we discovered that organic acids (malic acid), triterpenoids (asiatic acid), and lipids (JA) were remarkably upregulated. Malic acid is utilized in a variety of plant metabolic pathways, and NADP-ME is the malic enzyme (ME) responsible for metabolizing malic acid (Fig. 4F). The production and synthesis of both these substances can kill plant pathogens, secrete defense compounds such as flavonoids and lignans, and supply energy for biosynthesis^57^. JA is a lipid phytohormone that is also important in plant biotic and abiotic stresses. Researchers have found that JA can induce the expression of plant defense genes^58^ and dramatically increase POD to defend against *F. oxysporum*^59^, agreeing with our study.

*F. oxysporum* infection-induced restructuring of the faba bean rhizosphere microbiota reveals a pathogen-driven ecological reprogramming. The dominance of *Proteobacteria* and suppression of *Acidobacteria* and *Actinobacteria* in infected plants align with a hypoxia-acclimated community and might be driven by root rot-induced anoxia and fermentative metabolism^60–62^. Notably, the enrichment of *Dechloromonas*, which is reported to be associated with nitrogen fixation and anaerobic respiration, suggests compensatory metabolic strategies to counterbalance pathogen-induced nutrient deprivation^63,64^. On the other hand, the decline in *Sphingomonas* and *Lysobacter*, known for their antagonistic activity against pathogens, implies a collapse in native biocontrol functions, facilitating Fusarium to gain the upper hand around the rhizosphere^65,66^.

Functional shifts in microbial gene categories, such as the increase in the abundance of histidine kinase in the infected group, highlight adaptive signaling mechanisms under stress, potentially modulating virulence or environmental sensing^67,68^. The pathogen produces a large amount of CWDEs to dismantle host cell walls for nutrient acquisition^69^. GHs are involved in the hydrolysis of cellulose and hemicellulose, thereby destroying the plant cell walls^70^. Carbohydrates are released during the hydrolysis process of plant cell walls, and these glycans are regarded by plant receptors as alarm signals that trigger immunity^69,71^. GTs play a crucial role in the assembly of plant cell walls and the biosynthesis of extracellular polymeric substances (EPS) of bacteria^72,73^. Fusarium infection induces rhizosphere hypoxia and environmental acidification, driving both host and microbiota to reinforce cell wall synthesis for stress adaptation. AAs encompass lytic polysaccharide monooxygenases (LPMOs) and redox enzymes critical for lignin deconstruction, which serve as pivotal elements within fungal redox metabolic networks^74^. LPMOs are regarded as key frontline weapons in the warfare between hosts and attackers, such as fungal^75^.

Fusarium-infected plants showed a decrease in rhizosphere fungi with a higher abundance of viruses, which indicates pathogen-induced shifts in rhizosphere microbial communities, emphasizing the importance of ecological interactions in disease progression. Fungal communities were more responsive than bacterial communities to changes in vegetation, and fungi were the first consumers of below-ground, plant-derived carbon^19^. The enhanced carbon fermentation pathways, driven by hypoxia from root rot and microbial oxygen consumption, generate acidic metabolites that acidify the rhizosphere. This acidification suppresses beneficial microbes but also creates a self-reinforcing niche for Fusarium. Concurrently, the upregulation of phosphate acquisition systems (e.g., *pstS* and *phoX*) and PTS genes in infected plants reflects intensified microbial competition for phosphorus. Therefore, the host likely exacerbates phosphorus starvation, compromising root integrity and disease-suppressive capacities. Notably, inhibiting assimilatory sulfate reduction coupled with elevated cysteine biosynthesis suggests a compensatory plant response to sulfur limitation caused by impaired root function. Cysteine, a critical precursor for glutathione and defense-related metabolites, may be prioritized to mitigate oxidative stress and pathogen invasion. However, this reallocation of sulfur resources could further destabilize plant-microbe metabolic synergy. Collectively, Fusarium exploits carbon fermentation, phosphate competition, and sulfur metabolic dysregulation to weaken host defenses and establish rhizosphere dominance, underscoring the ability of pathogens to manipulate both host physiology and microbial ecology for virulence.

**Fig. 8:**
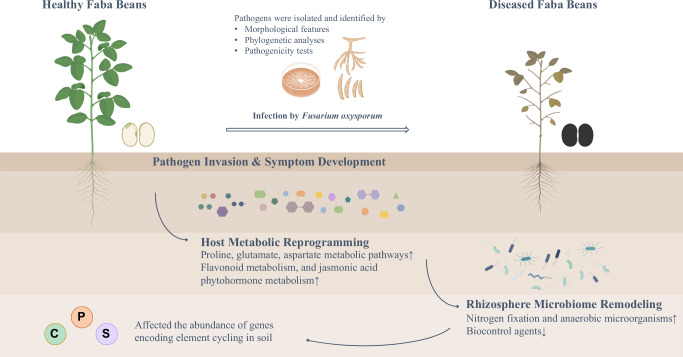
Pathogenic mechanisms and host defense in faba beans infected with *Fusarium oxysporum* causing Fusarium wilt.

Based on morphological characteristics, sequence analysis, and pathogenicity tests, we determined that *F. oxysporum* was responsible for FWD on faba bean (Fig. 8). Untargeted metabolomics analysis showed substantial variations in metabolites between healthy plants and diseased groups based on PCA score plot, PLS-DA score plot, and volcano plot. Faba beans activate defense responses to pathogenic fungal stress through coordinated metabolic reprogramming, as evidenced by marked alterations in key pathways such as arginine, aspartate, and glutamate metabolism, the TCA cycle, and JA signaling. We also reveal the relationship between the microbiome of the faba bean rhizosphere and Fusarium infection and describe the plant-microbiome-pathogen dynamics to further elucidate the impact and response of endophytes to plant disease infestation. *F. oxysporum* reshapes both taxonomic and functional landscapes of the rhizosphere to entrench its ecological niche and propagate disease progression. Our study not only provides a systematic identification of the causal agent but also discloses pathogenesis biomarkers of FWD and the mechanism of plant defense response, thus establishing a reliable basis for the prevention and management of *F. oxysporum*.

## Methods

### Sample collection, fungal isolation, and morphological identification

From 2021 to 2024, the occurrence of faba bean Fusarium wilt was estimated as 30–80% in Beibei (29°37’–30°05’ N, 106°18’–106°40’ E) and Rongchang District (29°15’–29°41’ N, 105°17’–105°44’ E) of Chongqing, China. These plants were grown in medium loam and gray-brown-purple soil, with a pH value of 6.5. Sampling was conducted during peak disease incidence periods, with three experimental fields selected per district. Within each field, three 20 m^2^ plots were randomly designated. The disease incidence, severity, and index were evaluated according to the study of Guo et al.^76^. The collected samples were transported to the Grassland Microbiome Laboratory, Southwest University, Chongqing, China.

Diseased tissues were cut into 5 mm^2^ pieces and flushed with tap water to remove dust and soil. Roots were disinfected with 75% ethanol for 10 s and 5% sodium hypochlorite for 1 min, and subsequently washed three times with sterile distilled water. Excess moisture was absorbed using autoclaved filter paper. The tissues were placed onto potato dextrose agar (PDA) supplemented with 0.1 g/L chloramphenicol. Cultures were maintained at 25 °C under complete darkness for 5–7 days. All isolates were purified from the hyphal margins and kept in PDA slants at 4 °C.

The pathogen was identified to genus level according to morphological traits. Isolates were cultured on PDA at 25 °C, and then 5-mm-diameter mycelia from the tip of the colony margins were placed in the center of each 90-mm-diameter Petri dish, with five replications per isolate. Colonies’ color and texture were evaluated by the naked eye and recorded. The size of 50 randomly selected conidia from each isolate was measured. Conidial shape and septation were recorded under a microscope (Nikon Eclipse E200, Shanghai, China).

### DNA extraction, PCR, and phylogenetic analyses

The isolates were cultured on PDA overlaid with cellophane and incubated at 25 °C for a week. Fresh mycelia of each strain were scraped from the surface. Total genomic DNA was extracted using the PlantGen DNA Kit CW0553A (Cwbio, Taizhou, China). PCR was carried out to amplify the internal transcribed spacer (ITS), beta-tubulin (TUB2), translation elongation factor 1-alpha (EF-1α), mitochondrial small subunit ribosomal DNA (mtSSU), second largest subunits of RNA polymerase II (rpb2), and glyceraldehyde-3-phosphate dehydrogenase (GAPDH) locus, based on primer pair ITS1/ITS4, BT2a/BT2b, EF1-728F/EF1-986R, NMS1/NMS2, fRPB2-5F/fRPB2-7cR, and GDF1/GDR1, respectively.

Each 50-mL-volume reaction mixture contained 21 mL of sterilized double-distilled water, 25 mL 2× PCR Taq Master Mix BN20009 (Biorigin, Beijing, China), 1 mL of each primer, and 2 mL template DNA. PCR was conducted in a thermal cycler (Bio-Rad T100, Guangzhou, China), and protocols were set as follows: an initial denaturing step of 5 min at 94 °C, followed by 35 cycles of denaturation at 94 °C for 30 s, annealing at 54 °C for 30 s and extension at 72 °C 90 s, and a final extension was performed at 72 °C for 10 min. The PCR results were validated by 1% agarose gel electrophoresis (Thermo Fisher PSC120M, Shanghai, China) and sequenced by Sanger’s method in GENEWIZ (Suzhou, China).

The consensus sequences were BLAST searched against the recognized sequences and the sequences of the ITS, TUB2, EF1-α, mtSSU, rpb2, and GAPDH genes for representative isolates DR and DW were deposited in the GenBank (http://www.ncbi.nlm.nih.gov). The phylogenetic tree comprising EF1-α and rpb2 regions was generated by MEGA 7 based on the Maximum-Likelihood method. The bootstrap values were assessed with 1000 replicates to test the branch strength.

### Pathogenicity tests

*F. oxysporum* strain DW was cultured on PDA at 25 °C for 7 days. Faba bean (*Vicia faba* L.) seeds of the “Tongchanxian” cultivar, sourced from the Institute of Silky Vegetables (Hechuan District, Chongqing, China), were used for pathogenicity assays. This cultivar represents the predominant faba bean genotype cultivated in Chongqing. For inoculum preparation, fungal cultures were gently rinsed with sterile distilled water to harvest conidia, and the conidial concentration was adjusted to 1 × 10^6^ conidia/mL. Faba bean seedlings, approximately 2 months of age, were cultivated in plastic pots. Pathogenicity tests were carried out on ten seedlings: seven as inoculated plants and three as the control group. Healthy roots of faba bean were immersed in conidial suspension for 30 min without wounds, while control plants received sterile water treatment. The experiment was repeated four times. All plants were maintained in a controlled-environment greenhouse under the following conditions: 28 °C daytime temperature, 60% relative humidity, and 12-h light/12-h dark photoperiod. The disease incidence and index in the pot experiment were recorded.

### Evaluation of antioxidant enzyme activities and membrane damage

Plant tissues (leaves and roots) were sampled from faba bean plants in the climate chamber 24 h after the onset of wilt symptoms with a disease severity of 3. The antioxidant functions in faba beans were evaluated according to the study of Zhang et al.^77^. Briefly, the activities of key antioxidant enzymes, including POD, SOD, and CAT, were measured using a spectrophotometer (Thermo Fisher Scientific, Shanghai, China). Chitinase activity was quantified using an assay kit (Solarbio Science & Technology Co., Ltd., Beijing, China) following the manufacturer’s instructions.

Membrane integrity was assessed through electrolyte permeability and MDA accumulation. Electrolyte leakage was measured using a DDS-11A conductivity meter (Shanghai Leici, Shanghai, China), and the MDA content was determined via the thiobarbituric acid (TBA) reaction.

### Metabolite extraction and derivatization

Faba beans were cultivated in an artificial climate chamber. As true leaves emerged, 1 × 10^6^ CFU/mL *F. oxysporum* spore suspension was irrigated on the base of faba beans. Roots and leaves samples were harvested 7–14 days post-inoculation, with Fusarium wilt severity of 3. Fresh plant samples were freeze-dried for 24 h and homogenized using a pulverizer machine (50 Hz). Powder samples weighing 50 mg were extracted with 0.6 mL of 70% methanol in sterile microcentrifuge tubes, followed by overnight incubation at 4 °C. Samples were subsequently sonicated for 5 min and then centrifuged at 12,000×*g* for 10 min. A Biosharp filter attached with a 0.22 μm membrane was used to filter the supernatant. For quality control (QC), 10 μL from each extract was pooled, with one QC analyzed per five samples to validate method stability.

LC-MS/MS analysis was conducted using a UHPLC system outfitted with a UPLC C18 column (Hypersil GOLD, 2.1 × 100 mm, particle size 1.9 μm, Thermo Fisher Scientific, USA) coupled with Q-Exactive Orbitrap (Thermo Fisher Scientific, USA). The injection volume was 2 μL with the column temperature at 35 °C and a flow rate of 0.2 mL/min. The composition of the mobile phase buffers and the parameters for both positive and negative ionization modes are detailed in Supplementary Tables 2 and 3.

The Q-Exactive Orbitrap mass spectrometer (Thermo Fisher Scientific, USA) was utilized to acquire MS/MS spectra in information-dependent acquisition (IDA) mode, controlled by Xcalibur software (Thermo Fisher Scientific, USA). The heated electrospray ionization (HESI) source parameters were optimized as follows: the capillary temperature was maintained at 320 °C; a full mass scan range of *m*/*z* 70–1050 was performed at a resolution of 70,000; and the sheath and auxiliary gas flow rates were set to 45 and 10 arbitrary units (Arb), respectively. For MS/MS analysis, a data-dependent acquisition (dd-MS2) mode was employed with a resolution of 35,000, and high-energy collision dissociation (HCD) was conducted at normalized collision energies (NCE) of 20, 40, and 60 eV. Ionization voltages were set to +3.5 kV (positive mode) and −2.8 kV (negative mode).

Raw data from the untargeted metabolomics analysis were processed using Compound Discoverer 2.1 software (Thermo Fisher Scientific, USA) for peak detection, extraction, deconvolution, normalization, and alignment. Metabolite identification was performed by matching against the mzCloud and mzVault databases. PCA and PLS-DA were conducted in SIMCA-P 14.1 (Umetrics, Umeå, Sweden). Differential metabolites were screened based on a VIP score > 1 and a *p* value < 0.05. Key metabolites selected for pathway analysis using MetaboAnalyst 5.0 (https://www.metaboanalyst.ca/).

### Metagenomic sequencing and analysis

Root samples were collected from both healthy and infected faba beans with severity of 3 to investigate pathogen-induced changes in the rhizosphere microbiome. Total genomic DNA was extracted from the root samples using the E.Z.N.A.® Soil DNA Kit (Omega Bio-tek, Norcross, GA, USA). The V3-V4 hypervariable regions of the bacterial 16S rRNA gene were amplified using the universal primer pair 338F (5′-ACTCCTACGGGAGGCAGCA-3′) and 806R (5′-GGACTACHVGGGTWTCTAAT-3′). The resulting amplicons were used to construct a sequencing library, which was then subjected to paired-end sequencing (2 × 250 bp) on an Illumina NovaSeq 6000 platform (Illumina, San Diego, CA, USA) at Biomarker Technologies Corporation (Beijing, China).

To ensure data quality, raw sequencing reads were first processed using Trimmomatic (v0.39) to remove low-quality bases and adapter sequences, yielding high-quality clean reads. Subsequently, Bowtie2 (v2.4.2) was employed to align and filter out reads mapping to the faba bean (*V. faba*) reference genome, thus eliminating host-derived sequences and retaining microbial reads for downstream analysis. De novo metagenomic assembly was performed using MEGAHIT (v1.2.9) with default parameters, and contigs shorter than 300 bp were discarded to ensure the reliability of the assembled sequences. The quality of the metagenomic assembly was assessed using QUAST (v5.0.2). Protein-coding genes were predicted from the assembled contigs using MetaGeneMark (v3.38) with default settings. To reduce redundancy, the predicted genes were clustered using MMseqs2 (v13.45111) with stringent parameters (95% sequence similarity and 90% coverage thresholds). The resulting non-redundant gene catalog was functionally annotated using multiple databases to assign taxonomic and functional information.

### Statistical analysis

Data analysis employed IBM SPSS Statistics 26.0 and GraphPad Prism 8.0 with an unpaired Student’s t-test and Duncan’s multiple range test. *p* < 0.05 was considered indicative of statistical significance. Bioinformatics analysis and visualization were conducted using the following online platforms: BMKCloud (https://www.biocloud.net), TBtools (https://github.com/CJ-Chen/TBtools-II/releases), and Weishengxin (https://www.bioinformatics.com.cn).

## Supplementary information

Supplementary Information

## Data Availability

Data will be made available on request.
