# Supplementary material for: Integrated metabolomics and metagenomics uncover pathogenic mechanisms of Fusarium wilt and faba bean defense responses
Source: NPJ Sci Food. 2026 Jan 19;10:25. doi: 10.1038/s41538-025-00673-8 (PMC12855188; doi:10.1038/s41538-025-00673-8)
Supplement: Supplementary file 1 — Supplementary Information [file 41538_2025_673_MOESM1_ESM.docx]

SUPPLEMENTARY MATERIALS

Effects of culture conditions on mycelia growth

Supplementary Table 1. Primers used in this study.

Supplementary Table 2. The mobile phase buffer composition for LC-MS/MS.

Supplementary Table 3. The elution gradient program of positive ion mode and negative ion mode using UHPLC-Q-Oritrap method.

Supplementary Table 4. *Fusarium* strains used in this study for phylogenetic analyses.

Supplementary Figure 1. Colony morphology on PDA, OA, RBCA, PCA, MEA, and PSA (from left to right).

Supplementary Figure 2. A permutation test plot (n = 200) for the PLS-DA model.

Supplementary Figure 3. A PERMANOVA test showing the variation between the healthy group (CK) and diseased group (F).

Supplementary Figure 4. Relative abundances of the bacterial community at the kingdom level collected from Fusarium-infected and healthy faba bean rhizosphere.

Supplementary Figure 5. The relative abundance of genes in the assimilatory sulfate reduction and cysteine biosynthesis pathway. * *p* < 0.05 and ns indicates not significant.

Supplementary Figure 6. Effects of temperature, pH, types of media, and light intensity on mycelial growth of *Fusarium oxysporum*. Error bars indicate the standard deviations based on three replicates. Different letters indicate significant differences among the diverse treatments.

**Effects of culture conditions on mycelia growth**

To prepare inoculum for exploring the effects of temperature, pH, culture medium, and light intensity on mycelial growth, *F. oxysporum* isolate DW were grown on PDA for 7 days. Excised from the colony edge of fungi, 4-mm-diameter mycelia were transferred to the middle of 90-mm-diameter plates of PDA. Cultures were incubated in the dark at different temperature regimes (15, 20, 22, 25, 28, 30 and 35 °C). To figure out the optimal pH value for the development of *F. oxysporum*, the pH of PDA was adjusted to 4, 5, 6, 7, 8, and 9 with 0.1 M NaOH or HCl, and plates were incubated at 25 °C in the dark. For determining the most suitable growth medium, a new batch of pathogens were inoculated on the plate center of PDA, Oatmeal Agar (OA), Potato Sucrose Agar (PSA), Malt Extract Agar (MEA), Plate Count Agar (PCA) and Rose Bengal Chloramphenicol Agar (RBCA) media and cultivated at 25 °C in the dark. As for the ideal illumination condition, pathogens were inoculated in PDA at 25 °C and light intensity was regulated as 0, 1000, 3000, 5000, and 7000 lx. There were four replicates per temperature, pH, medium type or light intensity. Radial growth was measured daily along two perpendicular diameters. Data analysis employed SPSS 16.0 and Excel 2016, with Duncan’s multiple range test for assessing the significance of difference.

The growth speed of *F. oxysporum* at different conditions was shown in Supplementary Figure 6. Colony on PDA showed optimal growth at 25 °C as the diameter was up to 74.14 ± 6.33 mm on the 5^th^ day. When the temperature was higher than 35 °C or lower than 15 °C, the growth rate of mycelia decreased significantly. *F. oxysporum* strains were able to grow in the pH range of 4 to 9. Mycelia grew most rapidly as the pH value reached 7. The morphological characterization of *F. oxysporum* on varied culture medium was shown in Supplementary Fig. 1. Colonies could spread over the 90 mm Petri dish in 7 days on PDA, PCA, OA and MEA medium and maximum radial growth of the pathogen was recorded on PDA, followed by PCA, on which the pathogen yielded a colony diameter of 60.11 ± 5.47 mm on the 5^th^ day. The mycelial growth rate was the slowest on PSA. The fastest growth rate was obtained in the dark, which indicates these two *F. oxysporum* strains prefer to grow in a complete darkness.

Supplementary Table 1. Primers used in this study.

| Locus | Primer | Primer sequence 5’ to 3’ |
| --- | --- | --- |
| Internal transcribed spacer (ITS) | ITS1 | TCCGTAGGTGAACCTGCGG |
|  | ITS4 | TCCTCCGCTTATTGATATGC |
| Beta-tubulin (TUB2) | BT2a | GGTAACCAAATCGGTGCTGCTTTC |
|  | BT2b | ACCCTCAGTGTAGTGACCCTTGGC |
| Translation elongation factor 1-alpha (EF-1α) | EF1-728F | CATCGAGAAGTTCGAGAAGG |
|  | EF1-986R | TACTTGAAGGAACCCTTACC |
| Mitochondrial small subunit ribosomal DNA (mtSSU) | NMS1 | CAGCAGTGAGGAATATTGGTCAATG |
|  | NMS2 | GCGGATCATCGAATTAAATAACAT |
| Second largest subunits of RNA polymerase II (rpb2) | fRPB2-5F | GAYGAYMGWGATCAYTTYGG |
|  | fRPB2-7cR | CCCATRGCTTGTYYRCCCAT |
| Glyceraldehyde-3-phosphate dehydrogenase (GAPDH) | GDF1 | GCCGTCAACGACCCCTTCATTGA |
|  | GDR1 | GGGTGGAGTCGTACTTGAGCATGT |

Supplementary Table 2. The mobile phase buffer composition for LC-MS/MS.

| Solvent | Composition |
| --- | --- |
| Solvent A | 0.1% formic acid in ultrapure water |
| Solvent B | 0.1% formic acid in methanol |
| Solvent C | 0.1% NH_3_ in ultrapure water |
| Solvent D | 0.1% NH_3_ in methanol |

Supplementary Table 3. The elution gradient program of positive ion mode and negative ion mode using UHPLC-Q-Oritrap method.

| **Positive ion mode** | | | | **Negative ion mode** | | | |
| --- | --- | --- | --- | --- | --- | --- | --- |
| t_R_ (min) | Flow velocity (ml· min^-1^) | A (%) | B (%) | t_R_ (min) | Flow velocity (ml· min^-1^) | C (%) | D (%) |
| 0-10 | 0.2 | 95 | 5 | 0-2.5 | 0.2 | 95 | 5 |
| 10-12 | 0.2 | 5 | 95 | 2.5-16.5 | 0.2 | 95 | 5 |
| 12-13 | 0.2 | 5 | 95 | 16.5-19 | 0.2 | 5 | 95 |
| 13.1-14 | 0.2 | 95 | 2 | 19-20 | 0.2 | 95 | 5 |

Supplementary Table 4. *Fusarium* strains used in this study for phylogenetic analyses.

| ***Fusarium* Species** | **Strain** | **Isolate Source** | **Location** | **EF1-α** | **rpb2** | **Reference** |
| --- | --- | --- | --- | --- | --- | --- |
| *Fusarium napiforme* | NRRL13604 | *Pennisetum typhoides* | Namibia | AF160266 | EF470117 | (O'Donnell et al., 2010) |
| *Fusarium nisikadoi* | RBG4043 | - | Australia | HQ667165 | HQ662692 | (Reis et al., 2023) |
| *Fusarium oxysporum* | NRRL34936 | *Solanum lycopersicum* | - | LS479646 | LS479200 | (Maryani et al., 2019) |
| *Fusarium oxysporum* | NRRL26406 | *Cucumis melo* | - | LS479647 | LS479201 | (Maryani et al., 2019) |
| *Fusarium oxysporum* | NRRL54002 | Soil | - | LS479640 | LS479194 | (Maryani et al., 2019) |
| *Fusarium oxysporum* | GR_FOAc | *Asparagus* sp. | Spain | MT305183 | MT305125 | (Brizuela et al., 2020) |
| *Fusarium oxysporum* | MA_FOA25 | *Asparagus* sp. | Spain | MT568947 | MT568979 | (Brizuela et al., 2020) |
| *Fusarium proliferatum* | GR_FP172 | *Asparagus* sp. | Spain | MT305208 | MT305150 | (Brizuela et al., 2020) |
| *Fusarium redolens* | NA_FRA02 | *Asparagus* sp. | Spain | MW091278 | MW091316 | (Brizuela et al., 2020) |
| *Fusarium dimerum* | NRRL36140 | Human blood | Netherlands | HM347133 | HM347218 | (O'Donnell et al., 2010) |
| *Fusarium mangiferae* | UMAF0924 | *Mangifera indica* | - | KP753402 | KP753442 | (Maryani et al., 2019) |
| *Fusarium aywerte* | RBG5743 | - | Australia | KP083250 | KP083278 | (Laurence et al., 2016) |
| *Fusarium commune* | NRRL28387 | *Dianthus caryophyllus* | Netherlands | AF246832 | JX171638 | (Baayen et al., 2000) |
| *Fusarium bulbicola* | NRRL13618 | *Nerine bowdenii* | Germany | KF466415 | KF466404 | (Laurence et al., 2016) |
| *Fusarium globosum* | NRRL26131 | *Zea mays* | South Africa | KF466417 | KF466406 | (Guo et al., 2021) |
| *Fusarium sacchari* | NRRL13999 | *Saccharum officinarum* | India | AF160278 | JX171580 | (Guo et al., 2021) |
| *Fusarium avenaceum* | FRC R-09495 | - | - | GQ915502 | GQ915486 | (Shang et al., 2018) |
| *Fusarium pseudograminearum* | RBG3580 | - | Australia | HQ667168 | HQ646400 | (Castillo et al., 2023) |
| *Fusarium culmorum* | RBG3558 | - | Australia | HQ667167 | HQ646401 | (Castillo et al., 2023) |

NRRL: Agricultural Research Service Culture Collection, Peoria, Illinois USA;

RBG: Royal Botanic Gardens Trust, Sydney, New South Wales, Australia;

UMAF: Microbiology and Plant Pathology Laboratory Collection, University of Malaga, Spain.

Supplementary Figure 1. Colony morphology on PDA, OA, RBCA, PCA, MEA, and PSA (from left to right).


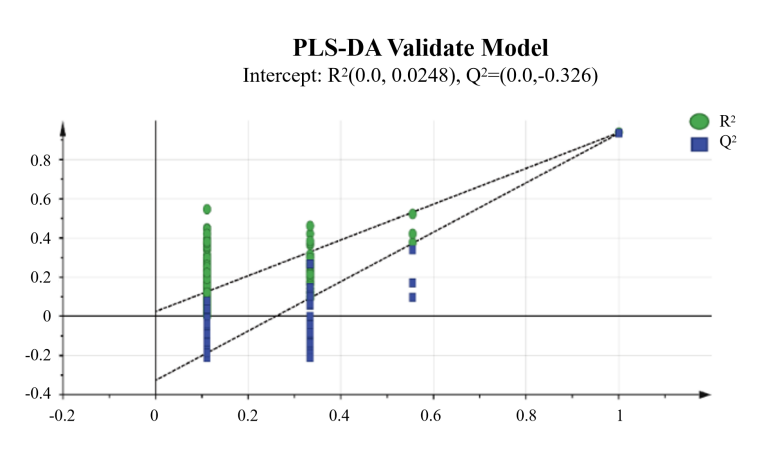


Supplementary Figure 2. A permutation test plot (n = 200) for the PLS-DA model.


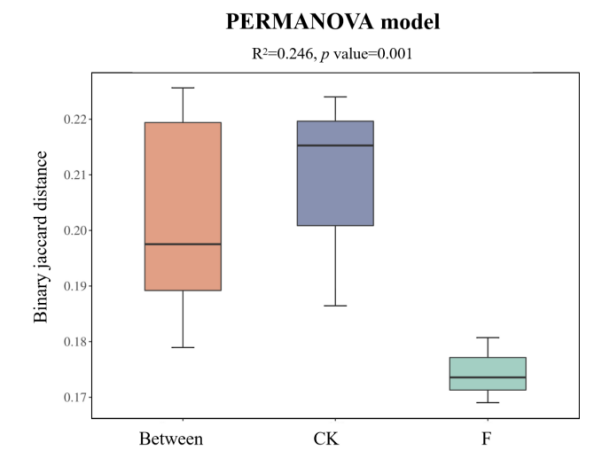


Supplementary Figure 3. A PERMANOVA test showing the variation between the healthy group (CK) and diseased group (F).

Supplementary Figure 4. Relative abundances of the bacterial community at the kingdom level collected from Fusarium-infected and healthy faba bean rhizosphere.


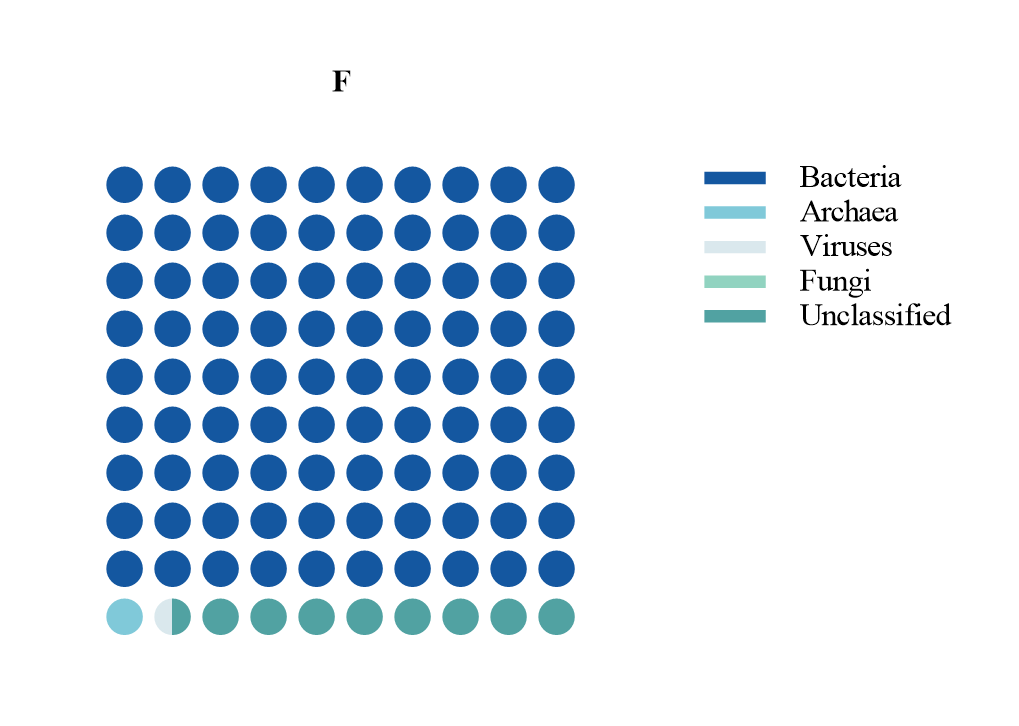

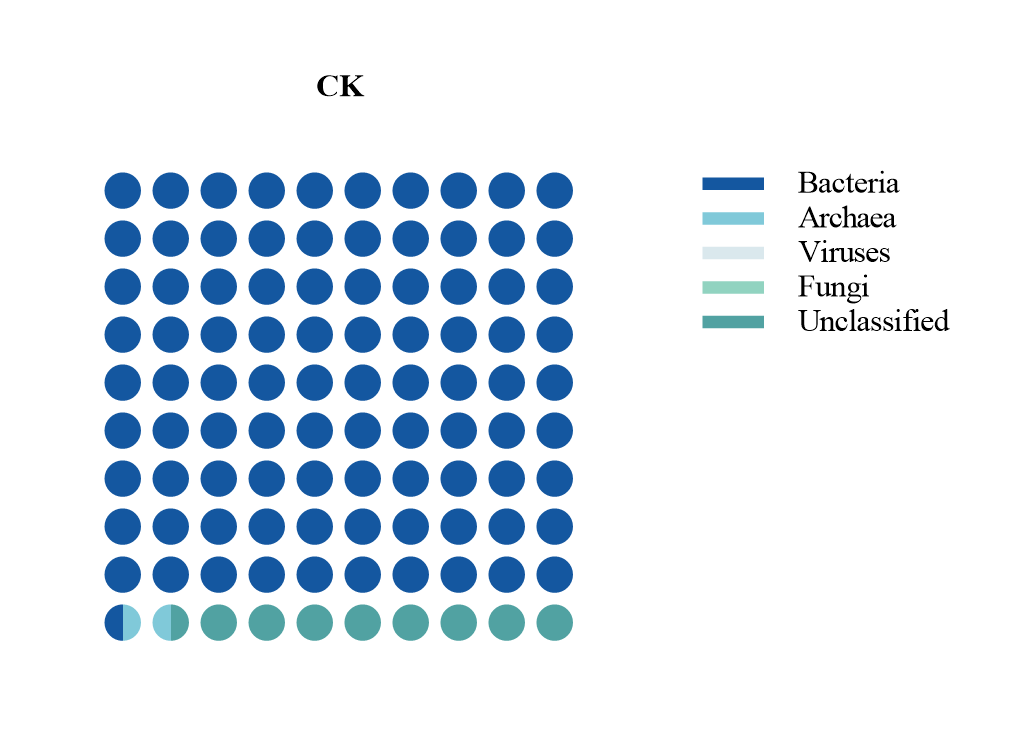


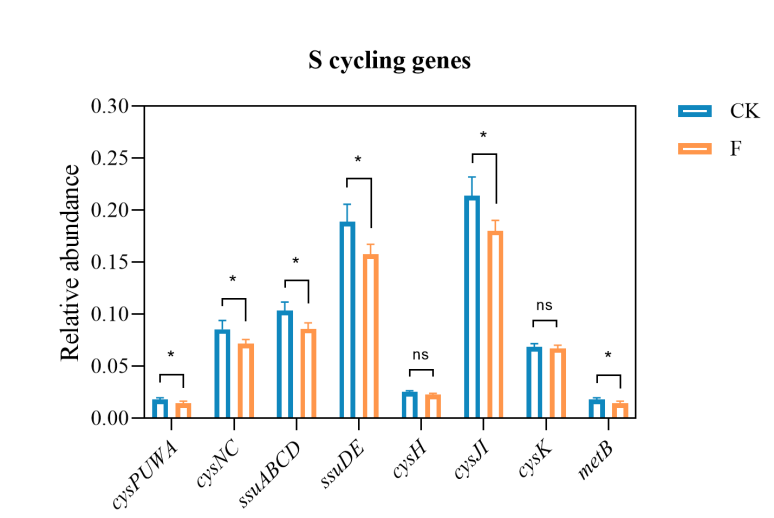


Supplementary Figure 5. The relative abundance of genes in the assimilatory sulfate reduction and cysteine biosynthesis pathway. * *p* < 0.05 and ns indicates not significant.


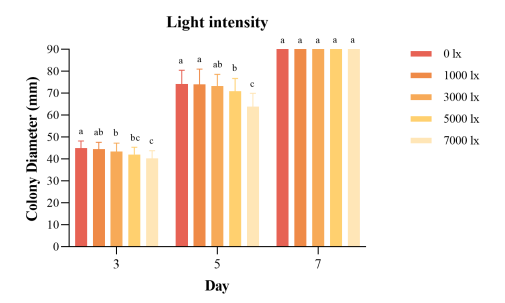

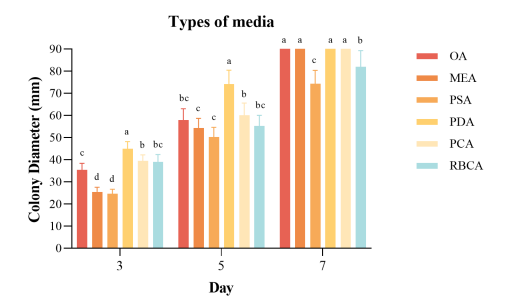

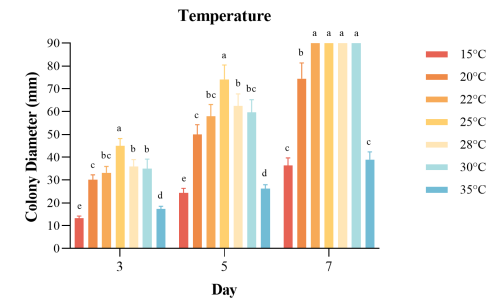

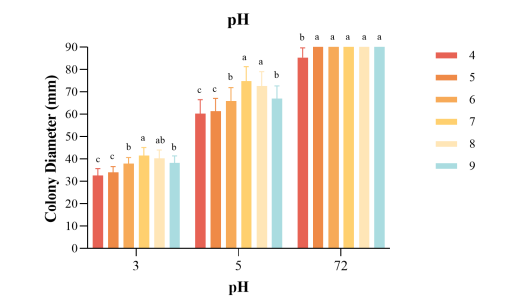


Supplementary Figure 6. Effects of temperature, pH, types of media, and light intensity on mycelial growth of *Fusarium oxysporum*. Error bars indicate the standard deviations based on three replicates. Different letters indicate significant differences among the diverse treatments.

**Reference**

Baayen RP, O'donnell K, Bonants PJM*, et al.*, 2000. Gene genealogies and AFLP analyses in the *Fusarium oxysporum* complex identify monophyletic and nonmonophyletic formae speciales causing wilt and rot disease. *Phytopathology* **90**(8), 891-900.

Brizuela AM, Lastra EDL, Marín-Guirao JI*, et al.*, 2020. Fusarium consortium populations associated with *Asparagus* crop in Spain and their role on field decline syndrome. *Journal of Fungi* **6**(4), 336.

Castillo SRM, Woodward S, Klopfenstein NB*, et al.*, 2023. Mycobiota associated with anthracnose and dieback symptoms on *Theobroma cacao* L. in Mérida State, Venezuela. *Summa Phytopathologica* **49**, DOI:10.1590/0100-5405/245874.

Guo ZN, Yu ZH, Li QL*, et al.*, 2021. *Fusarium* species associated with leaf spots of mango in China. *Microbial Pathogenesis* **150**, 104736.

Laurence MH, Walsh JL, Shuttleworth LA*, et al.*, 2016. Six novel species of Fusarium from natural ecosystems in Australia. *Fungal Diversity* **77**, 349-366.

Maryani N, Lombard L, Poerba YS*, et al.*, 2019. Phylogeny and genetic diversity of the banana Fusarium wilt pathogen *Fusarium oxysporum* f. sp. *cubense* in the Indonesian centre of origin. *Studies in Mycology* **92**, 155-194.

O'donnell K, Sutton DA, Rinaldi MG*, et al.*, 2010. Internet-accessible DNA sequence database for identifying Fusaria from human and animal infections. *Journal of Clinical Microbiology* **48**(10), 3708-3718.

Reis EM, Reis AC, Carmona M, 2023. Anthesis in small grains and Fusarium head blight infection. *Summa Phytopathologica* **49**, DOI: 10.1590/0100-5405/268456

Shang QJ, Phooicamsak R, Camporesi E*, et al.*, 2018. The holomorph of *Fusarium celtidicola* sp nov from *Celtis australis*. *Phytotaxa* **361**(3), 251-265.
